# The use of surgical glue and sutures in the aponeurosis synthesis of the abdominal wall in Wistar rats

**DOI:** 10.1590/0102-67202025000012e1881

**Published:** 2025-06-27

**Authors:** Carlos Alberto Lima UTRABO, Cesar Roberto BUSATO, Adriana Yuriko KOGA, Janete MACHOZEKI, Mirian GUIMARÃES, Danilo BELTRAME, Elcio MACHINSKI, Leandro Cavalcante LIPINSKI

**Affiliations:** 1 Universidade Estadual de Ponta Grossa, Department of Medicine – Ponta Grossa (PR), Brazil.

**Keywords:** Abdominal Wall, Aponeurosis, Suture Techniques, Parede abdominal, Aponeurose, Técnicas de Sutura

## Abstract

**Background:**

Adequate closure of the abdominal wall determines the success of the surgery.

**Aims:**

The aim of this study was to study the healing of the abdominal wall of rats by comparing the use of surgical glue (2-octyl cyanoacrylate) with polypropylene 3.0 thread and poliglecaprone 3.0 thread.

**Methods:**

A total of 60 Wistar rats were divided into two groups: Group 30 and Group 90. Each group was subdivided into three subgroups, surgical glue subgroup (C1), polypropylene subgroup (C2), and poliglecaprone subgroup (C3). An incision was made in the aponeurosis of the abdominal wall while maintaining the integrity of the parietal peritoneum. The 3 subgroups of 10 animals were euthanized on the 30th and 90th postoperative days. The abdominal wall fragments were submitted to macroscopic, histological, and tensiometric analysis.

**Results:**

Macroscopic analysis did not show any abnormality. Tensiometry on the 30th postoperative day showed a mean rupture tension of 30.98N in subgroup C1, 27.90N in subgroup C2, and 23.90N in subgroup C3. On the 90th postoperative day, the mean rupture tension was 30.05N in subgroup C1, 44.42N in subgroup C2, and 34.78N in subgroup C3.

**Conclusions:**

The synthesis of the abdominal aponeurosis performed with surgical glue (2-octyl cyanoacrylate) showed adequate resistance to rupture tension to maintain its integrity when compared with the synthesis with polypropylene thread or poliglecaprone thread, with both methods being equally effective.

## VISUAL ABSTRACT

**Figure:**
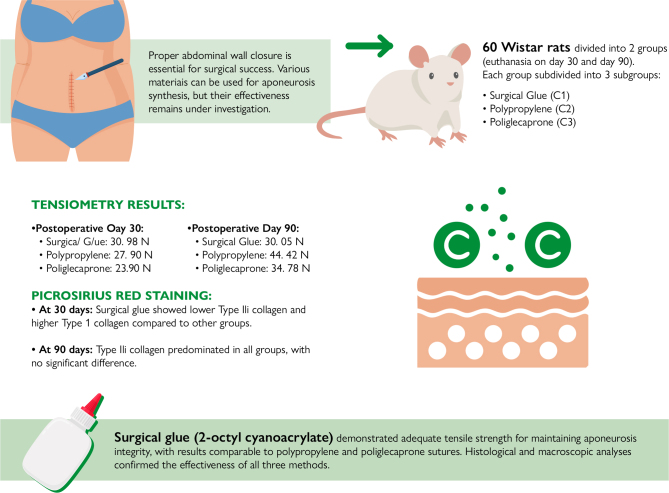

## RESUMO

**Racional:**

O fechamento adequado da parede abdominal determina o sucesso da cirurgia.

**Objetivos:**

Estudar a cicatrização da parede abdominal de ratos, comparando o uso de cola cirúrgica (2-octil cianoacrilato), com fio de polipropileno 3.0, e com fio de poliglecaprone 3.0.

**Métodos:**

Sessenta ratos Wistar foram divididos em 2 grupos: Grupo 30 e Grupo 90. Cada grupo foi subdividido em 3 subgrupos, subgrupo cola cirúrgica (C1); subgrupo polipropileno (C2); subgrupo poliglecaprone (C3). Foi realizada uma incisão na aponeurose da parede abdominal, mantendo a integridade do peritônio parietal. Os 3 subgrupos de 10 animais foram eutanasiados no 30º e 90º dias de pós-operatório. Os fragmentos da parede abdominal foram submetidos à análise macroscópica, histológica e tensiométrica.

**Resultados:**

A análise macroscópica não demonstrou nenhuma anormalidade. A tensiometria no 30º dia de pós-operatório mostrou uma tensão média de ruptura no subgrupo C1 de 30,98N, no subgrupo C2 27,90N e no subgrupo C3 23,90N. No 90º dia de pós-operatório, o subgrupo C1 apresentou uma tensão média de ruptura de 30,05N, o subgrupo C2 44,42N e o subgrupo C3 34,78N.

**Conclusões:**

A síntese da aponeurose abdominal realizada com cola cirúrgica (2-octil cianoacrilato) apresentou resistência adequada à tensão de ruptura para manter sua integridade, quando comparada à síntese com fio de polipropileno ou fio de poliglecaprone, sendo ambos os métodos igualmente eficazes.

## INTRODUCTION

The use of sutures for closing the abdominal wall dates back to 3,500 B.C. in Egypt, and this method remains the most common approach for approximating the edges of a surgical incision. Suture material is selected based on the biological properties of tissues and the physical and biological characteristics of the sutures and the structures to be sutured^7^. Ideal suture qualities include appropriate tensile strength, minimal tissue reaction, nontoxicity upon degradation, infection resistance, low friction coefficient, retention of strength for the necessary duration, nonallergenicity, non-mutagenicity, and adequate elasticity^1^.

Numerous comparative studies investigating the best method for abdominal wall synthesis are found in the literature. Some studies have compared the use of surgical glue for synthesis in the skin and aponeurosis, especially in mesh fixation for hernia repair^3,15^.

Depending on the type of suture material used, the exudative phase of the healing process may be prolonged. In this regard, synthetic sutures are generally more favorable as they result in a shorter and less intense inflammatory response. Cotton and silk sutures, on the other hand, cause a more intense tissue reaction^11^.

The healing of cutaneous and aponeurotic incisions is highly relevant in surgical practice, particularly regarding the kinetics of tensile strength gain and the final elongation of the incision. In general surgery, vertical midline incisions along the linea alba are frequently used, with a herniation rate ranging from 1 to 10%^6^.

Various techniques for tying surgical knots for anastomoses and laparotomy wound suturing are currently known. However, surgeons commonly continue to use traditional approaches, such as continuous suturing with braided nonabsorbable sutures. This approach has been associated with a high incidence of postoperative complications, including eventration and surgical site infections^13^.

Despite significant advancements in surgical techniques, equipment, and supplies, complications following abdominal wall synthesis remain a persistent issue. An ideal abdominal wall closure must be resistant and serve as a barrier against infection. It should have a low incidence of dehiscence, infection, hernia formation, and incisional pain, with minimal overall complications. The healing of abdominal incisions follows a similar process to that of other wounds. The inflammatory phase lasts approximately four days, followed by the proliferative phase for six weeks. Wound maturation continues for over a year, with abdominal wall tensile strength reaching approximately 80% of its original strength between the 6th and 20th postoperative weeks^18^.

Incisional hernia is a frequent complication of abdominal surgery. The estimated risk of developing an incisional hernia after a midline incision is 5.2%, but in high-risk patients, this incidence increases to 30%. Risk factors include a body mass index (BMI) greater than 25 kg/m^2^, the presence of an abdominal aortic aneurysm, and congenital connective tissue disorders. The technique for abdominal wall closure following a midline incision typically involves suturing with monofilament sutures, either continuous or interrupted. The European Hernia Society recommends closing the abdominal wall with slow-absorption sutures^16^.

Biological sealants, such as fibrin sealants, have a long history in surgery, initially used for sealing and hemostasis. Acrylic adhesives or cyanoacrylates belong to a highly interesting category of substances for clinical application. These adhesives are synthesized as monomers through the condensation of cyanoacetate with formaldehyde in the presence of catalysts, forming an adhesive film that rapidly polymerizes (within 5–60 s) when exposed to hydroxyl groups on the surface to be bonded. As an alternative for mesh fixation in hernia repair, the use of n-butyl-2-cyanoacrylate has been studied in rats^9,10^.

In 1949, Ardis was the first to synthesize cyanoacrylate, successfully exploring its strong adhesive properties. Its application in medicine and interest in surgery emerged before 1960, attracting the attention of surgeons as a potential adhesive substance for closing various tissues efficiently and rapidly^3^.

Cyanoacrylates are synthetic adhesives that polymerize quickly upon contact with water or blood^14^. They provide strong adhesion, low cost, bactericidal properties, and good cosmetic results. These characteristics make cyanoacrylates an attractive choice for abdominal wall closure or as a hemostatic agent in combination with traditional surgical closure techniques. Octyl α-cyanoacrylate induces minimal inflammation and demonstrates high adhesive capacity^4^.

The main goal of suturing is to approximate tissues without excessive tension, minimizing ischemia and tissue injury. Sutures are classified as absorbable or nonabsorbable. Absorbable sutures are derived from mammalian collagen, which is digested by bodily enzymes, or from synthetic polymers, which undergo hydrolysis. In the hydrolysis of synthetic sutures, water penetrates the suture filament, breaking down its polymer structure. To balance rapid absorption and prolonged tensile strength, sutures undergo chemical treatments that slow their degradation. The degradable material is eventually surrounded by fibroblasts, leading to fibrotic encapsulation. Sutures can be categorized as natural (e.g., mammalian tissues) or synthetic (e.g., polyglactin, poliglecaprone 25, and polydioxanone). Poliglecaprone 25 is absorbed within 90–120 days, maintaining predictable tensile strength for 21–28 days, offering high strength, low memory, minimal tissue trauma, and hydrolysis-based absorption^2,5,12^.

This study aims to compare polypropylene sutures, poliglecaprone sutures, and surgical glue in abdominal wall synthesis in rats, with tensiometric and histological evaluations on the 30th and 90th postoperative days.

The specific objectives are to evaluate the healing of abdominal wall incisions in rats by comparing the use of surgical glue (2-octyl cyanoacrylate), poliglecaprone 25 sutures, and polypropylene sutures, focusing on macroscopic evolution, tensiometry, healing process, and histology.

## METHODS

The study was conducted at the Laboratory of Operative Technique and Experimental Surgery of the Universidade Estadual de Ponta Grossa (UEPG). The research was approved by the Ethics Committee on Animal Use (CEUA) (Process nº. 21.000028198-0, Protocol UEPG nº. 0598220/2021).

The sample size (n) was calculated using G Power software. A total of 60 male Wistar rats, aged three months and weighing between 280 and 300 grams, were used. The animals were obtained from the UEPG animal facility and divided into two groups of 30 rats: Group 30 (n=30), euthanized on the 30th postoperative day, and Group 90 (n=30), euthanized on the 90th postoperative day. Each group was further divided into three subgroups: surgical glue (n=10), using surgical glue for aponeurosis synthesis; polypropylene (n=10), using 3-0 polypropylene sutures with separate stitches for aponeurosis closure; and poliglecaprone (n=10), using 3-0 poliglecaprone sutures with separate stitches. Each animal within the subgroups was assigned a number from 1 to 10.

### Preoperative care

The animals were housed in the UEPG animal facility under constant light and temperature conditions, with 12 h of light and 12 h of darkness at a temperature of 23°C, in a noise-free room illuminated with fluorescent lamps. They were kept in standard cages lined with wood shavings and were fed a commercial diet (Nuvital®) with access to treated water. The rats underwent a 12-h preoperative fasting period with access to water.

### Anesthesia

Anesthesia was performed via intraperitoneal administration of a mixture of 2% xylazine (0.4 mg/100 g body weight) and ketamine hydrochloride (10 mg/100 g body weight). The rats were considered anesthetized when they were unconscious and unresponsive to handling.

### Surgical procedures

Following anesthesia, the animals were placed in the supine position and secured by their limbs on the surgical table. Abdominal trichotomy was performed, followed by antisepsis with 10% PVPI (Iodopovidone, Rioquímica®) and placement of sterile surgical drapes. A paramedian supraumbilical abdominal incision of approximately 5 cm was made. An incision in the aponeurosis was performed while preserving the integrity of the parietal peritoneum.

**Figure 1:**
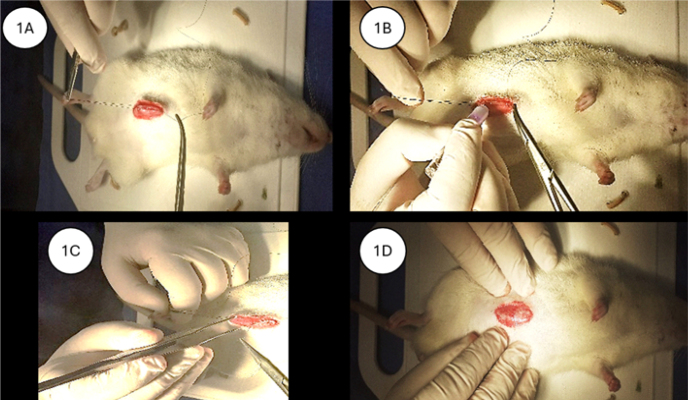
(A–D) Methods for using surgical glue in aponeurosis synthesis.

In the surgical glue subgroup, aponeurosis closure was performed using 2-octyl cyanoacrylate-based surgical glue, with approximation of the incision edges using three polypropylene stitches for initial alignment, followed by glue application along the incision line and compression of the wound edges with anatomical forceps. After the glue had set, the alignment stitches were removed (Figures 1A–D). In the polypropylene subgroup, aponeurosis closure was performed using separate “X” stitches with 3-0 polypropylene sutures, spaced approximately 0.5 cm apart, and placed 0.5 cm from the wound edge. In the poliglecaprone subgroup, aponeurosis synthesis was performed using separate “X” stitches with 3-0 poliglecaprone sutures. Skin closure was performed using a continuous intradermal suture with 4-0 mononylon.

### Postoperative care

The animals were kept in individual cages and warmed with artificial light until they fully recovered from anesthesia, at which point they could walk and feed. Postoperative analgesia was administered immediately using intramuscular fentanyl citrate every 12 h for the first 24 h, followed by oral paracetamol at a dose of 20 drops per 500 ml of drinking water. After recovery, the animals were transferred to an identified cage according to their group, where the date of the procedure and the synthesis material used in the aponeurosis closure were recorded (four rats per cage). Water and food were reintroduced 6 h postoperatively. Each animal underwent daily postoperative clinical evaluation, with careful inspection of the surgical wound.

### Euthanasia and sample collection

Euthanasia was performed on the 30th and 90th postoperative days via intraperitoneal injection of an overdose of ketamine combined with xylazine. A “U”-shaped incision was made in the skin and subcutaneous tissue, followed by reflection of the abdominal wall to expose the parietal peritoneum. Macroscopic evaluation of the wound and peritoneal cavity was conducted to assess the presence or absence of hematoma, infection, suture dehiscence, and adhesions (Table 1). Each of these parameters was classified as absent (0) or present (1).

**Table 1:** Macroscopic evaluation criteria.

Parameter	Evaluation
**Hematoma**	Absent–0
	Present–1
**Infection**	Absent–0
	Present–1
**Suture dehiscence**	Absent–0
	Present–1
**Adhesions**	Absent–0
	Present–1

If adhesions were present, they were classified and graded using the following methodology^8^: 1=minimal (released by delicate blunt dissection), 2=moderate (released by aggressive blunt dissection), and 3=intense (released only by sharp dissection). The mean adhesion index was calculated as the arithmetic mean of the values for each rat in each group.

**Figure 2:**
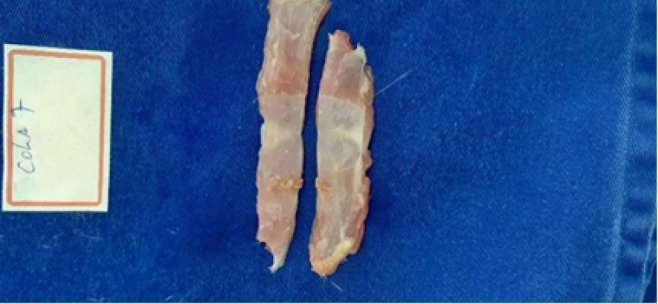
The excised abdominal wall, divided into cranial and caudal segments.

The excised abdominal wall, removed via a “U”-shaped incision, was divided by a median transverse cut into two segments: cranial and caudal (Figure 2). The cranial segments were stored in chilled saline, separately labeled, and subjected to tensiometric testing on the same day as euthanasia. The caudal segments were placed in 10% formalin solution, separately labeled, and stored for later histological analysis.

### Tensiometric analysis

Tensiometry was performed using a Shimadzu AG-I tensiometer (Japan) with Trapezium 2 software. The device was programmed to record tissue area and thickness data and the results obtained from the test. The tests were conducted at room temperature (24°C), with the device calibrated to a speed of 50 mm/min, and the results were expressed in Newtons (N). Each cranial segment was measured with a caliper and then fixed to the tensiometer via the muscle tissues adjacent to the suture site.

### Histological analysis

For histological analysis, specimens were sectioned with a microtome into 5-micrometer slices. Slides were stained with hematoxylin-eosin (HE) and picrosirius red and then examined by a pathologist blinded to the animal groups. All HE-stained slides were analyzed for the presence of foreign body granulomas, inflammatory response, and fibrosis. Picrosirius red-stained slides were used to quantify type 1 and type 3 collagen using ImageJ software.

The inflammatory parameters were analyzed quantitatively using the methodology of Vizzoto Junior et al^17^. The study was based on the number of foci per field, which were characterized by the presence of neutrophils, edema, congestion, mononuclear cells, granulation tissue, and fibrosis (Table 2). The data were classified as severe (-3), moderate (-2), mild (-1), and absent (0).

**Table 2:** Classification and index assignment for histological findings in hematoxylin-eosin staining^17^.

Inflammatory parameters	Severity			
	Severe	Moderate	Mild	Absent
Neutrophils	-3	-2	-1	0
Edema	-3	-2	-1	0
Congestion	-3	-2	-1	0
Mononuclear cells	3	2	1	0
Granulation tissue	3	2	1	0
Fibrosis	3	2	1	0

The classification of the inflammatory process phase was based on the sum of the indices found in each subgroup, with the following score ranges: -9 to -3 indicating an acute phase, -2.9 to 3 indicating a subacute phase, and 3.1 to 9 indicating a chronic phase. Each score represented the cumulative indices observed in each subgroup, as detailed in Table 3.

**Table 3:** Characterization of the inflammatory process phase according to the final score of each animal^17^.

Sum of indices in each subgroup	Final classification score
Acute	-9 to -3
Subacute	-2.9 to 3
Chronic	3.1 to 9

For the analysis of variance in tensiometry, inflammatory process evaluation across different materials, and fibrosis, nonparametric statistical tests were applied. To compare the results, the Kolmogorov–Smirnov normality test and Student’s T-test were performed. A significance level of 0.05 (5%) was established for rejecting the null hypothesis.

## RESULTS

### Tensiometry

**Figure 3:**
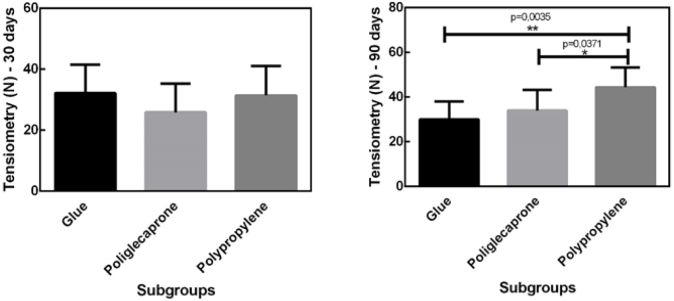
Mean rupture tension of the tissues on the 30th and 90th postoperative day.No statistically significant difference was found in the tensiometric analysis when comparing the surgical glue and poliglecaprone subgroups, the latter being fully absorbed by the end of 90 postoperative days (p>0.05).

The mean rupture tension of the tissues was 30.98 N in the surgical glue subgroup, 23.90 N in the poliglecaprone subgroup, and 27.90 N in the polypropylene subgroup on the 30th postoperative day, while on the 90th postoperative day, the values were 30.05 N, 34.78 N, and 44.42 N, respectively (Figure 3).

### Macroscopic evaluation

No animal presented hematoma, adhesions, infection, fistula, suture dehiscence, or incisional hernia. The wound edges were completely approximated in all animals.

### Microscopic evaluation

**Figure 4:**
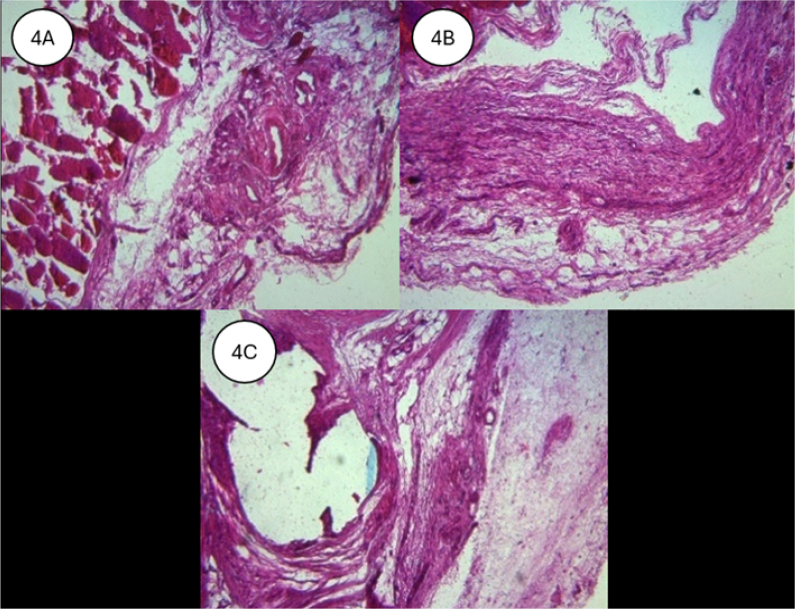
(A) Surgical glue group (HE 40X). B) Complete absorption of the poliglecaprone suture on the 90th day. C) Presence of the polypropylene suture surrounded by fibrotic tissue.

HE staining at 20X magnification demonstrated that all tissues were in the subacute inflammatory phase, with no statistical difference between subgroups. Complete absorption of the poliglecaprone suture was observed on the 90th day, while polypropylene sutures remained surrounded by fibrotic tissue (Figure 4).

**Figure 5:**
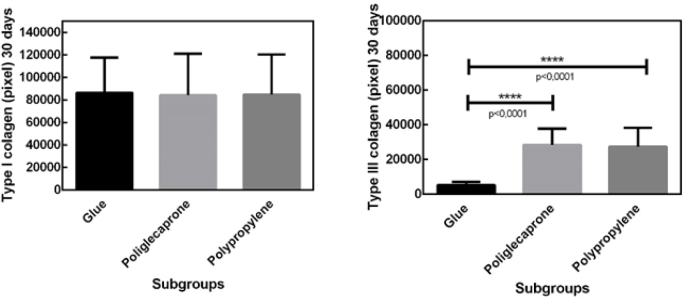
Picrosirius staining on the 30th postoperative day showing a lower quantity of type III collagen in the surgical glue subgroup compared to the other subgroups and a higher quantity of type I collagen, with no significant differences between subgroups.

Picrosirius staining on the 30th postoperative day showed a lower quantity of type III collagen in the surgical glue subgroup compared to the other subgroups and a higher quantity of type I collagen, with no significant differences between subgroups (Figure 5).

**Figure 6:**
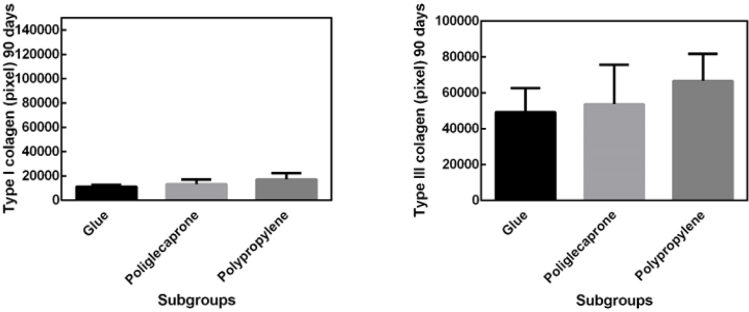
Predominance of type III collagen over type I collagen on the 90th day, with no significant differences between subgroups.

On the 90th day, there was a predominance of type III collagen over type I collagen, with no significant differences between subgroups (Figure 6).

### Phases of the inflammatory process

The inflammatory process phase on both the 30th and 90th postoperative days, analyzed using the method by Vizzoto Junior et al.^17^, was classified as subacute (Tables 4 and 5).

**Table 4:** Final score for the 30-day group – subacute phase.

Group	Final score	Phase
Poliglecaprone	0.6	Subacute
Surgical glue	0.5	Subacute
Polypropylene	0.5	Subacute

**Table 5:** Final score for the 90-day group – subacute phase.

Group	Final score	Phase
Poliglecaprone	1.4	Subacute
Surgical glue	0.7	Subacute
Polypropylene	0.7	Subacute

## DISCUSSION

The aponeurosis is a laminar structure whose function is to withstand forces, protecting body segments in their position. Its histological structure consists of fibrous tissue maintained by collagen, with a small amount of extracellular matrix and cells, providing strength to its structure and low elasticity. The abdominal wall serves as an entry point for intra-abdominal procedures and, in some cases, for retroperitoneal access. The wide variety of materials and techniques available for abdominal wall closure has drawn significant interest from surgeons^3^.

Most studies involving cyanoacrylate for tissue synthesis in animal models focus on skin closure. When compared to traditional sutures, cyanoacrylate adhesives offer several advantages, including shorter application time, effective approximation of wound edges, immediate hemostasis, reduced inflammation, and absence of infection^8^.

A review of the literature reveals no specific studies on the use of surgical glue for aponeurosis synthesis, except for the work of Batista et al.^3^, who utilized N-butyl-2-cyanoacrylate.

In the present study, tensiometry of the synthesized tissues demonstrated that, on postoperative day 30, the mean rupture tension was 30.98N for surgical glue, 23.90N for poliglecaprone, and 27.90N for polypropylene. On postoperative day 90, the values were 30.06N for surgical glue, 35.40N for poliglecaprone, and 44.42N for polypropylene.

On postoperative day 30, no statistically significant difference was observed in the mean rupture tension among the subgroups. However, on postoperative day 90, polypropylene exhibited greater resistance compared to the other subgroups, while no significant difference was found between the surgical glue and poliglecaprone subgroups. It is noteworthy that poliglecaprone is fully absorbed by postoperative day 90.

Studies indicate that the maximum intra-abdominal pressure exerted in humans, such as during coughing or jumping, reaches 16N/cm, representing the highest force to which the abdominal wall is subjected^8^.

In a morphological evaluation, Batista et al. observed that the two materials analyzed in their study (3-0 nylon sutures and N-butyl-2-cyanoacrylate) did not exhibit significant differences in the healing process of the anterior abdominal wall aponeurosis in rats. The wound closure with the tissue adhesive was faster, and greater tensile strength was observed in the adhesive group 14 days after the initial surgery^3^.

Findings from the present study indicate that, in tissues where surgical glue was used, the mean rupture tension was 30.98N on postoperative day 30 and 30.05N on postoperative day 90 — values well above the maximum intra-abdominal pressure exerted during coughing or jumping (16N). This demonstrates that the use of surgical glue ensures adequate and secure maintenance of the abdominal wall’s integrity, with results comparable to the tensile strength of poliglecaprone sutures, which are fully absorbed by postoperative day 90. The resistance of 2-octyl-cyanoacrylate synthesis is evident in both groups analyzed in this study.

Utrabo et al., using the same 2-octyl-cyanoacrylate glue in small amounts for mesh fixation in abdominal wall defect repair in rats, found that tensiometric analysis revealed rupture tension values sufficient to maintain the integrity of the repaired wall^15^.

The mean rupture tension results in this study confirm the efficacy and feasibility of using surgical glue for aponeurosis synthesis.

## CONCLUSIONS

The synthesis of the abdominal aponeurosis using surgical glue (2-octyl cyanoacrylate) demonstrated adequate tensile strength to maintain its integrity when compared to synthesis with polypropylene and poliglecaprone sutures, proving that the methods are equally effective.

## Data Availability

The datasets generated and/or analyzed during the current study are available from the corresponding author upon reasonable request.
